# Metallodrugs: Mechanisms of Action, Molecular Targets and Biological Activity

**DOI:** 10.3390/ijms23073504

**Published:** 2022-03-23

**Authors:** Giarita Ferraro, Antonello Merlino

**Affiliations:** Department of Chemical Sciences, University of Naples Federico II, Complesso Universitario di Monte Sant’Angelo, Via Cinthia 21, I-80126 Naples, Italy

The research interest in the field of inorganic medicinal chemistry had a large increase after the serendipitous discovery of the cytotoxic activity of cisplatin by Rosenberg at the end of 1960s [1]. Since then, cisplatin has entered clinical practice and become one of the most common treatments for solid tumors. Unfortunately, the use of cisplatin and its derivatives is associated with undesired side effects, such as general toxicity and intrinsic and acquired drug-resistance [2]. For these reasons, alternative metallodrugs based on non-Pt metals have been synthetized, characterized and tested for biological activity in recent years [3,4,5]. Although, initially, DNA seemed the exclusive target for metallodrugs [6], successive studies have revealed that various metabolites, peptides and proteins have a central role in the recognition, transport and mechanism of action of these compounds [7,8,9]. Thus, a deeper understanding of the molecular bases of the interaction of metallodrugs with these molecules is needed.

The aim of this Special Issue was to collect computational and experimental data on the biological activity of well-established and novel metallodrugs, in addition to information on the interactions of metals and metal-based drugs with metabolites, nucleic acids, peptides and proteins. Herein, the 11 original articles published in this Special Issue are summarized and discussed in the frame of recent advances in the field.

Four manuscripts submitted for this Special Issue report new data on the cytotoxic and potential antitumor actions of Pt(II), Fe(III), Cu(II), Zn(II) and Ir(III) compounds [10,11,12,13].

Annunziata et al. describe the synthesis, characterization and cytotoxic activity of square-planar cationic Pt(II) complexes containing glucoconjugated triazole ligands [10]. The results obtained with the newly synthetized compounds were compared to those found using parent five-coordinate (5C) complexes bearing the same triazole ligands [14]. The square-planar species were more stable and less cytotoxic than the corresponding 5C compounds, but they exhibited a certain selectivity. These results suggest that the stability of Pt compounds is important for preserving their performance as cytotoxic agents, and support the hypothesis that coordinative saturation can be a point in favor of their biological action [15].

In the paper by Marchetti and coworkers, the in vitro anticancer activity of piano stool mononuclear and binuclear Ir(III) complexes based on the pentamethylcyclopentadienyl ligand (Cp*) was evaluated [11]. Various compounds were synthetized, characterized and assessed for their cytotoxicity against six human and rodent cancer cell lines. The results of the study indicated that the 2-phenylpyridyl (PhPy) mononuclear derivatives [Ir(η^5^-C_5_Me_4_H)(k_N_,k_C_PhPy)Cl] and [Ir(η^5^-C_5_Me_4_(4-C_6_H_4_F))(k_N_,k_C_PhPy)Cl] were the most active molecules among those under investigation. They showed good selectivity, were inactive towards healthy cells, and had a triple effect on cancer cells. Their cytotoxic activity was reached through proliferation inhibition, apoptosis activation and senescence induction. This latter effect is significant to note, since it is rarely observed for organoiridium complexes.

Verreault and coworkers investigated the cytotoxic activity of six iron-based ferrocifens against 15 glioblastoma patient-derived cell lines. The authors found that the studied molecules showed an IC_50_ value ranging from nanomolar to micromolar [12]. The results provide new information on the mechanism of action of ferrocifens, and indicate that differences in the chemical structures of these molecules significantly alter their behavior against brain cancer.

Klein and coworkers extensively characterized three complexes of Fe(III), Cu(II) and Zn(II) bearing the redox active, non-innocent ligand opo (opo = 9-oxido-phenalenone) by ^1^H nuclear magnetic resonance, electron paramagnetic resonance, UV-vis absorption spectroscopy and electrochemical analysis [13]. They assessed their stability in different organic solvents and tested their cytotoxic activity. They found that the compounds showed antiproliferative activity against colon and breast cancer cell lines in the micromolar range [13].

The work by Szefler et al. presents a computational study on the affinity of carboplatin to B vitamins, aiming to establish if the vitamins—as components of beet and carrot juice—could possibly lead to a reduction in the efficacy of this drug. Their data indicated that carboplatin can bind vitamins B3 and B6, and their computational prediction was also verified by experimental data [16].

Hemphill and coworkers investigated the potential cellular and molecular targets of a trithiolato-bridged arene ruthenium complex conjugated to 9-(2-hydroxyethyl)-adenine [17], which inhibits the protozoan parasites *Toxoplasma gondii* and *Trypanosoma brucei*. Transmission electron microscopy (TEM) images revealed structural alterations of parasite mitochondria after a few hours’ exposure to the drug. Furthermore, Ru complex molecular targets were analyzed by differential affinity chromatography coupled to shotgun–mass spectrometry, and a mitochondrial ATP-synthase subunit was identified as the main Ru complex target. Altogether, these results demonstrated that the trithiolato-bridged arene ruthenium complex can interfere with key steps of cellular metabolism.

The use of dinuclear trithiolato-bridged arene ruthenium complexes as drugs in vivo is limited by their scarce solubility in water. An attempt to overcome this limitation and increase their selectivity for cancer cells was carried out by encapsulating the trithiolato-bridged arene ruthenium complex Diruthenium-1 within a horse spleen apo-ferritin (hsAFt) nanocage [18]. hsAFt was also used to encapsulate Au, Pt, Ru and heterobimetallic compounds [19].

The same approach was also used to encapsulate arsenoplatin-1 (AP-1) within hsAFt [20]. AP-1 is a dual-action anticancer metallodrug with a promising pharmacological profile that features the simultaneous presence of a cisplatin-like center and an arsenite center [21,22]. The adduct formed upon the encapsulation of AP-1 within hsAFt was structurally characterized (Figure 1A): AP-1 binds the side chain of His49 upon the release of the chloride ligand (Figure 1B). The adduct was less toxic than the free drug, but more selective, since the concentration needed to kill cancer cells is half that needed to kill immortalized cells [20].

Structural data have been also collected on adducts formed upon the reaction of a model protein hen egg white lysozyme with the paddlewheel dirhodium tetraacetate complex (Rh_2_(act)_4_, act = acetate) under different experimental conditions [23]. This compound exhibited in vivo anticancer activity against Ehrlich ascites, L1210 tumors, sarcoma 180 and P388 leukemia [24,25,26]. The structures revealed that Rh_2_(act)_4_ degrades under the investigated conditions, at a variance with that observed upon the reaction of the same compounds with other proteins [27]. Dimeric Rh-Rh units and monomeric fragments mainly bind the protein close to His, Asp and Lys side chains. The work, which describes rare examples of structures of Rh/protein adducts [28], demonstrates that Ru_2_(act)_4_ reacts with lysozyme differently from the analogous Ru_2_(act)_4_ complex [29] and from RhCl_3_ [30].

Moreover, the interaction of metal ions and metal complexes with peptides is also distinctly important. Three papers in the Special Issue report studies based on metals or metal compounds and peptide recognition processes.

D. Marasco and coworkers studied the possible use of three Pt(II) compounds (bearing 1, 10-phenathroline, 2, 2′:6′, 2″-terpyridine (terpy) and 2, 2′-bipyridine) as inhibitors of the aggregation of the β-amyloid (Aβ) peptide Aβ_21–40_ [31]. The misfolding of Aβ, its aggregation, the formation of fibrils and deposition in the brain all play major roles in the development of Alzheimer’s disease [32]. The investigated compounds inhibited Aβ_21–40_ aggregation and modulated the peptide disaggregation via the formation of adducts where the metal center loses chloride ligands. The compound with terpy induced the formation of soluble monomeric forms of Aβ_21–40_ and reduced the toxicity of the peptide in human SH-SY5Y neuroblastoma cells. These results pave the way for the future application of metal-based drugs in the treatment of neurodegenerative diseases.

La Mendola and coworkers studied the interaction between copper and different peptides [33,34], including: a peptide derived from the N-terminal domain of Angiogenin (Ang) [33]; a ribonuclease essential for angiogenesis stimulation [35]; and peptides belonging to the N-terminal domain of nerve growth (NGF) [34], a member of the neurotrophin family that is essential for neuron survival [36]. They demonstrated that sequence 1–17 of Ang is involved in copper uptake and that the acetylation of the terminal amino group of the peptide decreases the intracellular metal level [33]. Since several diseases are characterized by an upregulated Ang expression together with an altered copper metabolism, these alterations can be used as potential targets for the design and development of specific therapies [37,38].

Furthermore, the stability constants of copper complex species formed with the dimeric forms of the sequence 1–15 of NGF have been studied [34]. At a physiological pH, NGF peptides bind copper ion with a higher affinity than N-terminal peptides of Aβ, and a lower affinity than that observed for peptides derived from human copper transporter 1 (hCtr1), the protein responsible for copper uptake in neurons. This interaction suggests NGF as a possible regulator of copper homeostasis in the synaptic space, which has a key role in the process of memory formation.

Altogether, these new studies show once again the importance of metals in regulating the activity and function of peptides, and the importance of studying metal compounds endowed with unique biological properties. The data suggest that the investigations on the design and development of new metal-based drugs will continue, since new therapeutic applications of metal complexes can be explored. We hope that, in the near future, we can gain a better understanding of these mechanisms of action, as well as of the targeting and activation strategies of these compounds; a deeper knowledge of these aspects will lead to future generations of metallodrugs with reduced side effects and a wider spectrum of activity.

## Figures and Tables

**Figure 1:**
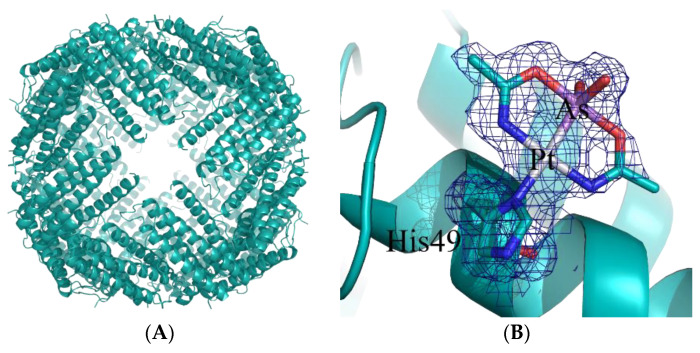
Cartoon representation of the overall structure of the hsAFt nanocage (**A**) and details of AP-1 binding site (**B**). An AP-1 moiety binds to the ND1 atom of His49. In panel (**B**), the 2Fo-Fc electron density map is colored in blue and contoured at 0.5σ.
